# Analysis of stress-induced duplex destabilization (SIDD) properties of replication origins, genes and intergenes in the fission yeast, *Schizosaccharomyces pombe*

**DOI:** 10.1186/1756-0500-5-643

**Published:** 2012-11-19

**Authors:** Mukesh P Yadav, Sreedevi Padmanabhan, Vishnu P Tripathi, Rahul K Mishra, Dharani D Dubey

**Affiliations:** 1 Department of Biotechnology, Veer Bahadur Singh Purvanchal University, Jaunpur, Uttar Pradesh 222001, India

**Keywords:** Replication origins, ARS elements, *S. pombe*, SIDD

## Abstract

**Background:**

Replication and transcription, the two key functions of DNA, require unwinding of the DNA double helix. It has been shown that replication origins in the budding yeast, *Saccharomyces cerevisiae* contain an easily unwound stretch of DNA. We have used a recently developed method for determining the locations and degrees of stress-induced duplex destabilization (SIDD) for all the reported replication origins in the genome of the fission yeast, *Schizosaccharomyces pombe*.

**Results:**

We have found that the origins are more susceptible to SIDD as compared to the non-origin intergenic regions (NOIRs) and genes. SIDD analysis of many known origins in other eukaryotes suggests that SIDD is a common property of replication origins. Interestingly, the previously shown deletion-dependent changes in the activities of the origins of the *ura4* origin region on chromosome 3 are paralleled by changes in SIDD properties, suggesting SIDD’s role in origin activity. SIDD profiling following *in silico* deletions of some origins suggests that many of the closely spaced *S. pombe* origins could be clusters of two or three weak origins, similar to the *ura4* origin region.

**Conclusion:**

SIDD appears to be a highly conserved, functionally important property of replication origins in *S. pombe* and other organisms. The distinctly low SIDD scores of origins and the long range effects of genetic alterations on SIDD properties provide a unique predictive potential to the SIDD analysis. This could be used in exploring different aspects of structural and functional organization of origins including interactions between closely spaced origins.

## Background

The duplication of genomic DNA in eukaryotic cells is accomplished by replication forks emanating bidirectionally from a large number of replication origins distributed throughout the genome [1,2]. Replication origins of *Saccharomyces cerevisiae,* the best characterized eukaryotic origins, are confined to specific DNA regions of 100–200 bp known as autonomously replicating sequences or ARS elements as the plasmids containing them can replicate autonomously in yeast cells [3,4]. The *S. cerevisiae* ARS elements are marked by the presence of two essential features, a close match to an 11 to15-bp ARS consensus sequence (ACS) and a stretch of easily unwound DNA next to ACS at the 3^′^ end of its T-rich strand [5-7]. The ACS is occupied by the origin recognition complex (ORC) and other initiator proteins [8] and the adjacent easily unwound region facilitates double helix opening for initiation of replication. Mutations in ACS that destroy ORC binding or in the easily unwound region that increase its stability also cause loss of origin activity [7]. Although the ACS is essential for origin activity, it is not sufficient and only a small fraction of all genomic ACSs (~500 out of ~12000 ACS matches) is associated with active origins in *S. cerevisiae.* However, its presence has been helpful in precisely locating origins in broad origin regions mapped in genome-wide studies [9-15]. Unlike *S. cerevisiae*, the replication origins of all other studied eukaryotic cells lack a conserved nucleotide sequence.

Replication origins have also been extensively studied in the fission yeast, *Schizosaccharomyces pombe*, where they correspond, mostly but not always [16], to ARS elements. *S. pombe* ARS elements are ~1 kb in size and they lack any known conserved nucleotide sequences [17] like other eukaryotic origins. In addition to 54 precisely localized origins by the two-dimensional agarose gel electrophoresis origin mapping technique (2D technique) and DNA combing [18-26], different genome-wide origin mapping studies have located several hundred to nearly one thousand potential origins in *S. pombe*[11,25,27-29]. All these origins are confined to the intergenic regions (IRs), which usually have higher AT content than the genomic average. Most of them are inefficient, firing only in a small fraction of a cell population, and the closely spaced origins seem to interact with each other in a hierarchical fashion [18]. Replication origins of *S. pombe* have not been extensively analyzed for their helical stability and the destabilization properties of the origin-containing intergenic regions (OIRs) remain to be known.

Of the three methods developed to analyze duplex destabilization of any given stretch of DNA, those used by MELTMAP [30] and WEBTHERMODYN [31] are based only on the local nucleotide composition while the one used by WEBSIDD [32] also takes into account the effects of superhelical stress occurring *in vivo* on strand opening behaviors of all the base pairs in a topologically constrained domain. The global coupling of strand opening behaviors results into widespread changes in destabilization properties of all the base-pairs in the domain and a deletion/mutation in one region may cause such changes in regions several kilo-base away from it [33]. The computation of the destabilization energy, *G*(x)*,* also called SIDD (stress-induced duplex destabilization) energy, using the WEBSIDD tool has been shown to predict accurately the location and extent of destabilization of different regulatory regions, promoters and replication origins, in viral, bacterial and yeast genomes and in some cases it has been found to be important for the origin activity [13,33-35]. The SIDD analysis results for *S. cerevisiae* origins have been found not only consistent with that of the duplex unwinding element (DUE) analysis by WEBTHERMODYN but also more informative in several ways because of its above-mentioned features [7,33].

In this study we have analyzed the SIDD profile of all known replication origins of *S. pombe* mapped previously using different techniques. Our results show that *S. pombe* origins are more susceptible to stress-induced duplex destabilization than their adjacent genes and non-origin intergenes and that, in case of closely spaced origins, the extent of destabilization appears to influence the origin activity.

## Methods

### Sequence data collection

A fixed window size of 5 kb, unless otherwise mentioned, was used for all origins which have been mapped earlier to relatively smaller regions using the 2D technique, bioinformatics [25] or the microarray methods [27,28] with the origins placed at the center. Origins larger than 5 kb were analyzed within the coordinates mentioned in the referred papers. Once boundaries were marked, sequences were downloaded from the *S. pombe* GeneDB, modified version of March-04-2011. The randomly selected comparison regions, genes and intergenes, were analyzed similarly.

### Calculation of SIDD profiles

We used WEBSIDD server [32] to determine SIDD profile of previously reported origins, genes and non-origin intergenes using fixed window sizes as mentioned above. The conditions for the assessment of superhelical denaturation and the basis of computation have been described (WEBSIDD manual).

## Results

**Figure 1:**
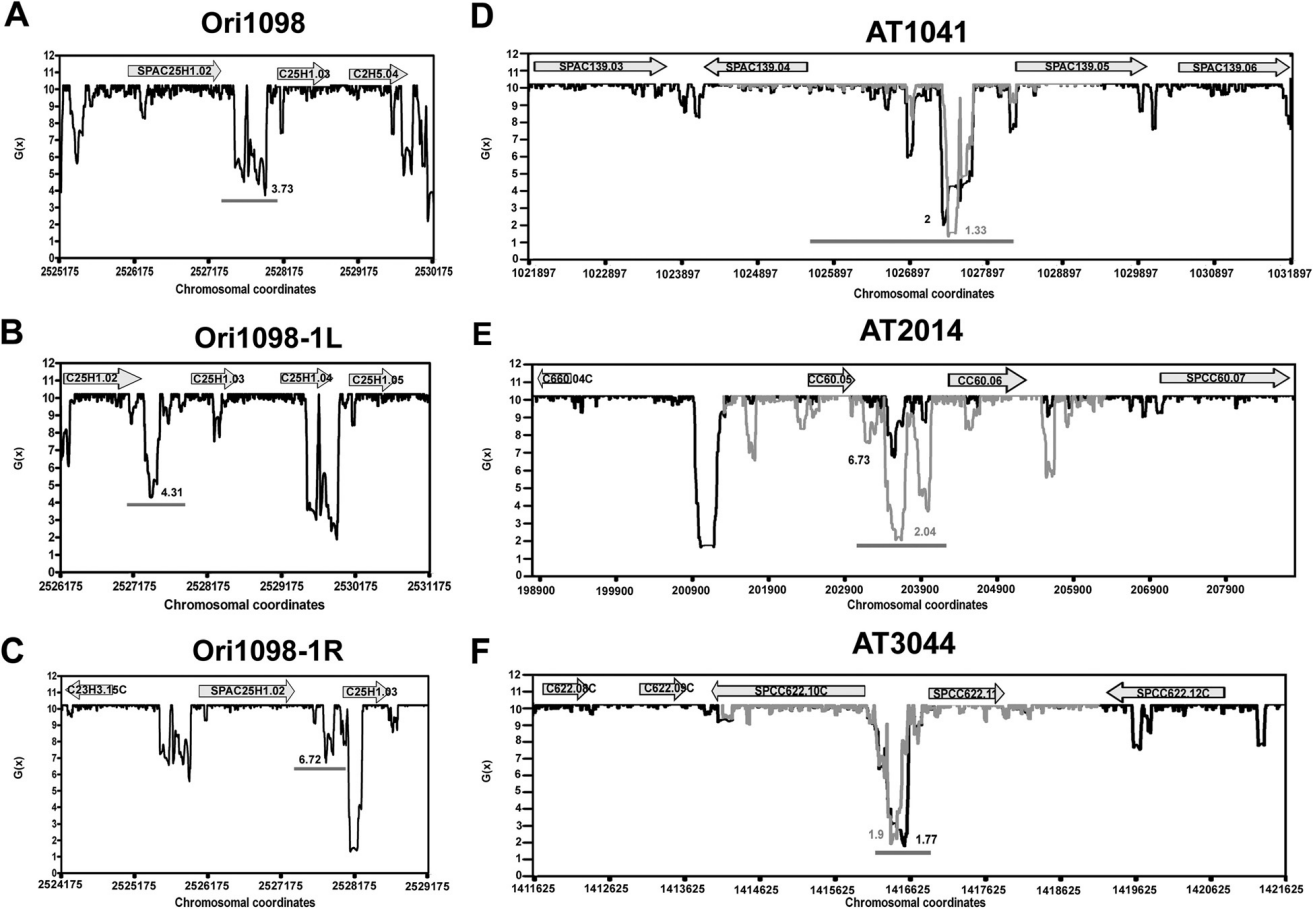
**Variability patterns of SIDD profiles in sliding and fixed windows.** (**A-C**) Variability of SIDD profiles of Ori1098 in sliding windows. Note that the deep SIDD valley corresponding to the origin (grey bar) in the center of a 5-kb window (**A**) becomes shallow on sliding 1 kb from left to right (**B**) and shallower on sliding 1kb from right to left (**C**). (**D-F**) The locations of the SIDD valleys show little or no alteration although their depths change to different extents between 5-kb and 10-kb windows centered on an origin.

The SIDD profiling using the WEBSIDD results in a plot between the destabilization energy, *G*(x), under specified growth conditions and degree of superhelicity, and base-pair position. Under these conditions, the base-pairs with a *G*(x) value close to 10 kcal/mol are as stable as they would be in a relaxed molecule and those with a *G*(x) value near 0 kcal/mol are highly destabilized. The *G*(x) values between 0–10 kcal/mol show positions of intermediate stability [33]. Even a partial destabilization of a DNA region caused by a few kcal/mol lowering of *G*(x) will significantly facilitate its opening by other factors like helicases. SIDD analysis of *S. cerevisiae* replication origins has shown earlier that they are highly susceptible to superhelically driven DNA duplex destabilization [33]. To find out whether the replication origins of *S. pombe* are similarly destabilized, we performed SIDD calculations of all major reported replication origins in this organism. Because the boundaries of the topologically constrained domains occurring *in vivo* are not known and because of the variability of SIDD profile from window to window and for different sized windows centered on the same origin (Figure 1), we thought it necessary to figure out the analysis parameters that would predict the origin locations most reliably. For this, we determined SIDD profiles of 48 replication origins detected earlier using a two-dimensional origin mapping technique [36] and DNA combing [22]. These gold standard techniques are capable of physically mapping replication origins to short stretches of DNA ranging from several hundred base-pairs to a few kilo-bases. Initially, we used sliding windows which produced extremely variable SIDD profiles and any given region appeared highly stable or unstable in different overlapping windows. This is probably due to the fact that in SIDD calculations the melting properties of different basepairs in a domain are influenced by each other, and, in a sliding window, the relative position of every basepair is altered. However when different sized windows were centered on the same region, the variations were greatly reduced, mostly confined to limited changes in the depths of the valleys and, sometimes, to their slightly shifted locations (Figure 1D-F). Table S1 shows the results obtained using one variable window, the OIR plus 1-kb flanking regions on both sides, and two fixed windows of 5 kb and10 kb, all centered on origins to determine SIDD profiles of all 48 2D origins. Although all the origins appeared to contain SIDD valleys in all 3 windows, the SIDD valleys generally appeared shallower with increasing window size (e.g. Figure 1E). The average lowest *G*(x) for the 48 origins in the variable window, 5-kb window and 10-kb window analyses was 1.58-, 1.65- and 2.56- kcal/mol, respectively. Within the 5-kb windows for these 48 2D-gel-verified origins, valleys bottoms had *G*(x) values between 1.23 and 2.48 kcal/mol (Additional file 1: Table S1). All further analysis was done using a fixed window of 5 kb unless otherwise mentioned.

**Figure 2:**
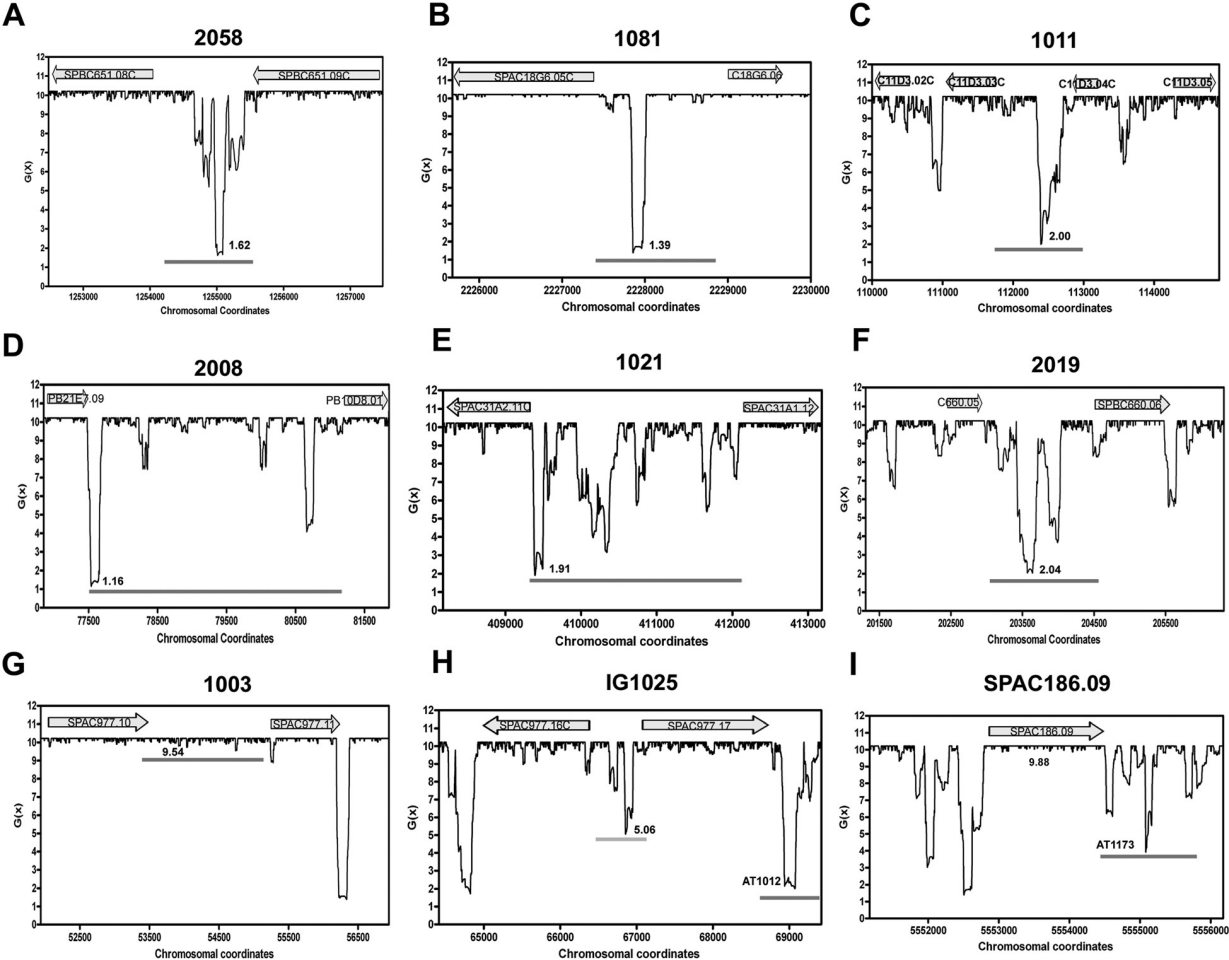
**Representative patterns of the SIDD profiles of different genomic entities.** (**A-G**) origins, (**H**) a NOIR and, (**I**) a gene. All these origins except (**D**) and (**G**) are 2D-gel proven. The locations of the origins/AT islands are marked with grey bars. Note in (**G**) the presence of a deep SIDD valley not overlapping but close to Ori1003, one of the four origins found to be highly stable in this study.

Next, we performed SIDD analysis of 355 AT islands [25], 460 origins mapped by high-resolution microarray ChIP-chip analysis of the locations of ORC and MCM subunits [28] and 476 weak origins mapped by copy number method [37]. Most of these origins are confined to relatively short stretches of DNA. Because the SIDD properties of AT islands and ChIP-chip origins were indistinguishable from each other and because most of them overlap with each other, we merged them together as 564 ‘origins’ for presentation (Additional file 2: Table S2). The 440 copy number origins which did not match with the origins are listed as weak origins. All the origins and weak origins showed the presence of one, two or sometimes three or more significantly destabilized IRs (Figure 2). All but four of the 564 origins (99.3%) were found to be highly unstable with the average minimum *G*(x) value 1.85 kcal/mol (Table 1, Additional file 2: Table S2). The four OIRs showing a minimum *G*(x) greater than 9.0 kcal/mol associated with Ori1003, Ori2024, Ori2030 and AT-1120, are all flanked by destabilized regions on one or both sides (see the profile of Ori1003 in Figure 2G). Among the ChIP-chip origins, the average lowest *G*(x) for the early origins (1.77 kcal/mol) differed only slightly from that of the late origins (1.88 kcal/mol). The 440 weak origins showed an average lowest *G*(x) value 2.46 kcal/mol. Similarly analyzed 360 genes appeared to be highly stable with an average lowest *G*(x) value 8.47 kcal/mol. NOIRs showed an intermediate level of stability with an average lowest *G*(x) value 3.16 kcal/mol for 434 intergenes. Using the 5-kb window analysis, 91% origins, 67% weak origins, 43% NOIRs and <2% genes coincided with the lowest SIDD valley in their window. The number of origins, genes and NOIRs studied, the averages of their lowest *G*(x) values and their AT contents are given in Table 1.

**Table 1:** Averages of the lowest SIDD values, sizes and AT contents of different entities

**Entities**	**Number analyzed**	**Average lowest *G*(x) value (kcal/mol)**	**Average size (bp)**	**AT content (%)**	**% of entities containing lowest *G*(x) point**
**ChIP - chip origins & AT islands (OIRs)**	576 (564)*	1.85	2284	69.98	91
**Weak origins (OIRs)**	440	2.46	1323	69.56	67
**NOIRs**	434	3.16	1536	67.93	43
**Genes**	360	8.47	1359	60.9	2

*564 SIDD values for 576 origins because 2 adjacent origins were considered as one, in 11 cases.

Averages of the lowest *G*(x) values, sizes and AT% of the OIRs, NOIRs and genes, and the % of these entities containing the lowest *G*(x) point in a 5-kb window centered on the entity.

**Figure 3:**
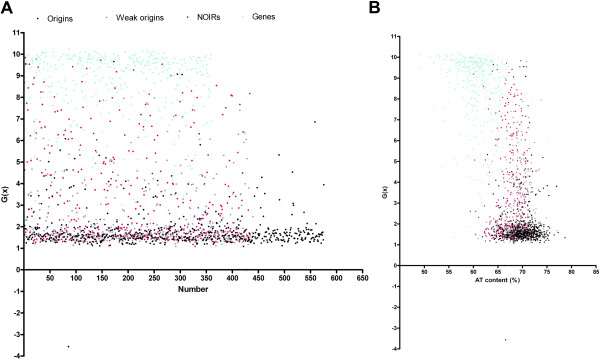
**Origins are more susceptible to SIDD than NOIRs and genes.** (**A**) Distribution patterns of the lowest *G*(x) values of 564 origins (ChIP-chip origins and AT-islands) [25,28] (black dots), 440 weak origins [37] (grey dots), 434 NOIRs (magenta dots) and 360 genes (cyan dots). (**B**) The graph of AT% vs lowest *G*(x) values of the four analyzed entities, ChIP-chip origins and ATislands (black dots), weak origins (grey dots), NOIRs (magenta dots) and genes (cyan dots) in the 5-kb context.

The distribution patterns of the lowest *G*(x) values of these entities are clearly distinguishable from each other (Figure 3A). The origins comprised a vast majority (>76% to >80%) of all the intergenes falling in any of the three arbitrarily fixed lowest *G*(x) cut off values of 2.0, 2.5 and, 3.0 kcal/mol (Table 2). Since all the genomic origin mapping studies, whose origin mapping data have been used for the present study, have failed to report at least 3 2-D proven origins, ori2, ori4 and ori5, in a 75-kb region [19], this ratio is likely to increase in favor of origins as future studies come up with additional origins.

**Table 2:** Majority of origins are more susceptible to SIDD than NOIRs and genes

***G*(x) cut off value (kcal/mol)**	**OIRs**		**Genes (360)**	**NOIRs (434)**	**% Intergenes (OIRs+NOIRs) predicted as origins**
	**Origins (564)**	**Weak origins(440)**			
2.0	470 (83.3%)	283 (64.3%)	5 (1.4%)	232 (53.5%, 39%*)	76.45 (80.41*)
2.5	503 (89.2%)	316 (71.8%)	6 (1.7%)	261 (60.1%, 48%*)	75.83 (80.27*)
3.0	516 (91.4%)	338 (76.8%)	6 (1.7%)	283 (65.2%, 52%*)	75.1 (79.68*)

*After excluding NOIRs that correspond to some of the recently reported origins [38].

Numbers of OIRs, genes, and NOIRs falling in the three lowest *G*(x) cut off categories. Their percentages are indicated in parentheses. Note that a vast majority (>76 to >80%) of the intergenic regions falling in these categories correspond to putative replication origins.

We used Wilcoxon-Mann–Whitney rank sum test to determine the statistical significance of the observed differences between the lowest *G*(x) values of OIRs and NOIRs and found that they differed significantly (*P*=<0.05) for both, the origins and the weak origins. The origins and the weak origins also differed significantly from each other (*P*=<0.05) in their *G*(x) values. We conclude that the susceptibility to stress-induced destabilization of different entities of the *S. pombe* genome shows the following pattern: origins>weak origins>NOIRs>genes.

**Figure 4:**
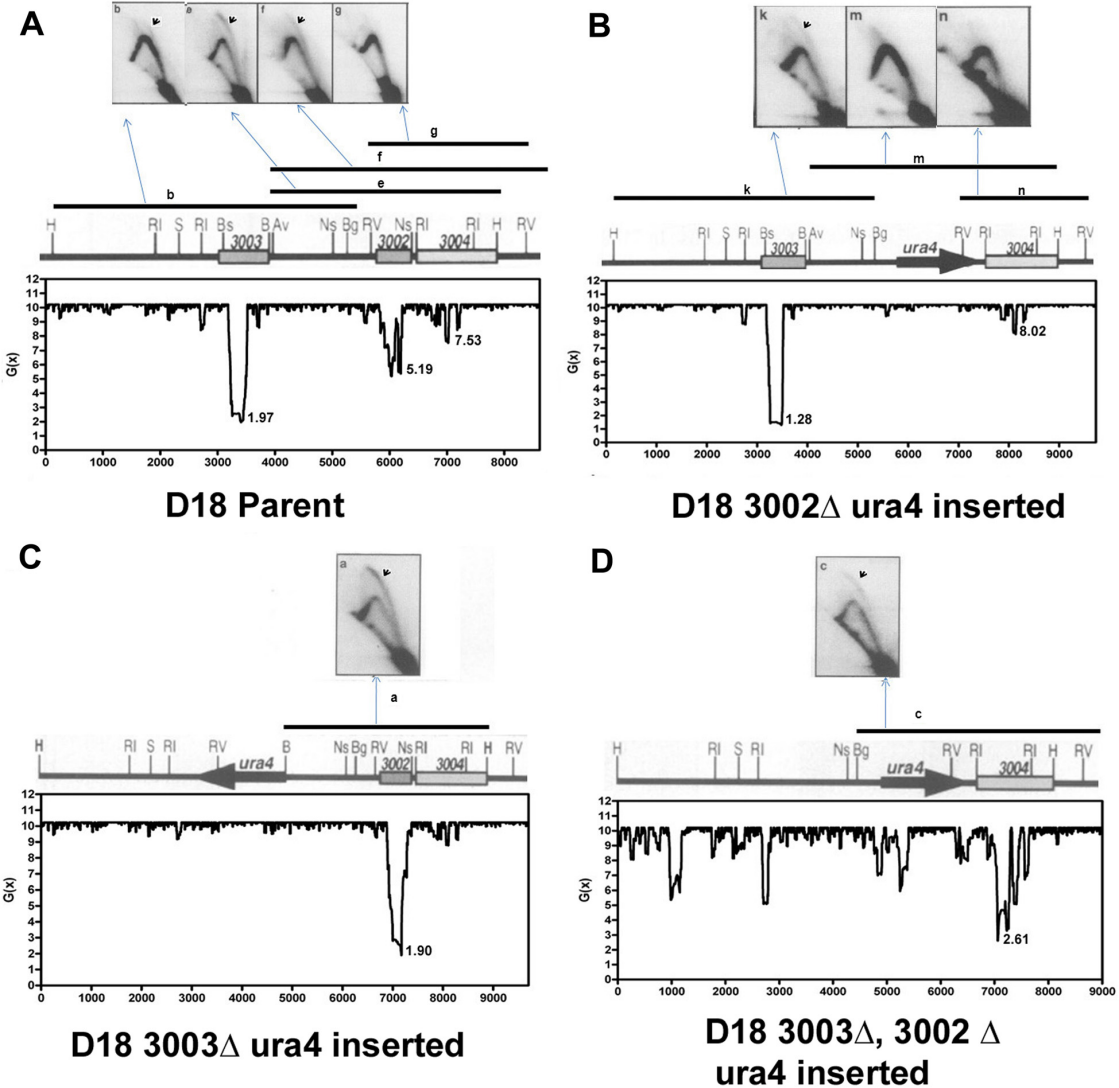
**Alterations in the origin activity are paralleled by SIDD changes in the *ura4 *origin region.** Relationship between the extent of SIDD and origin activity in the *ura4* origin region in (**A**) wild-type, and, (**BD**) ARS deletion *S. pombe* strains [18]. The map of the concerned region, locations of ARS elements, restriction sites (Av=AvrII, B=BamHI, Bg=BglII, Bs=BstBI, C=ClaI RI=EcoRI, RV=EcoRV, H=HinDIII, N=NruI, Ns=NsiI,S=SmaI) and the 2D autoradiograms of different fragments shown on the top of each section have been reproduced from [18] with permission. The arrows mark the bubble arc signal, indicative of the origin activity of the detected fragment. The lowest *G*(x) value for each ARS element is shown in kcal/mol near the valley.

To find out the functional significance of this coincidence, we performed SIDD analysis of the *ura4* origin region [18], one of the best genetically dissected chromosomal origin regions in *S. pombe*. This origin region contains two strong ARS elements, *ars3002, ars3003* and one weak ARS element, *ars3004,* which later turned out to be a part of extended *ars3002*[39], within a ~5-kb region on chromosome 3. Each of the two strong ARS elements functions as a replication origin in the chromosome. When any one of them is deleted the origin activity seems to be transferred to the other. When both of them are deleted, only then the weak ARS, *ars3004*, becomes active as origin [18]. The SIDD profile of the region showed that both the chromosomally active origins, *ars3002* and *ars3003,* were stress-destabilized in the wild type cells (Figure 4A) and deletion of either of them resulted into the disappearance of the corresponding SIDD valleys and formation of deeper valleys under the remaining ARS (Figure 4B and C). Deletion of both of them resulted into destabilization of *ars3004* which had been shown to get activated as an origin in this strain (Figure 4D). These results strongly suggested that the extent of SIDD could be an important factor influencing the origin activity.

**Figure 5:**
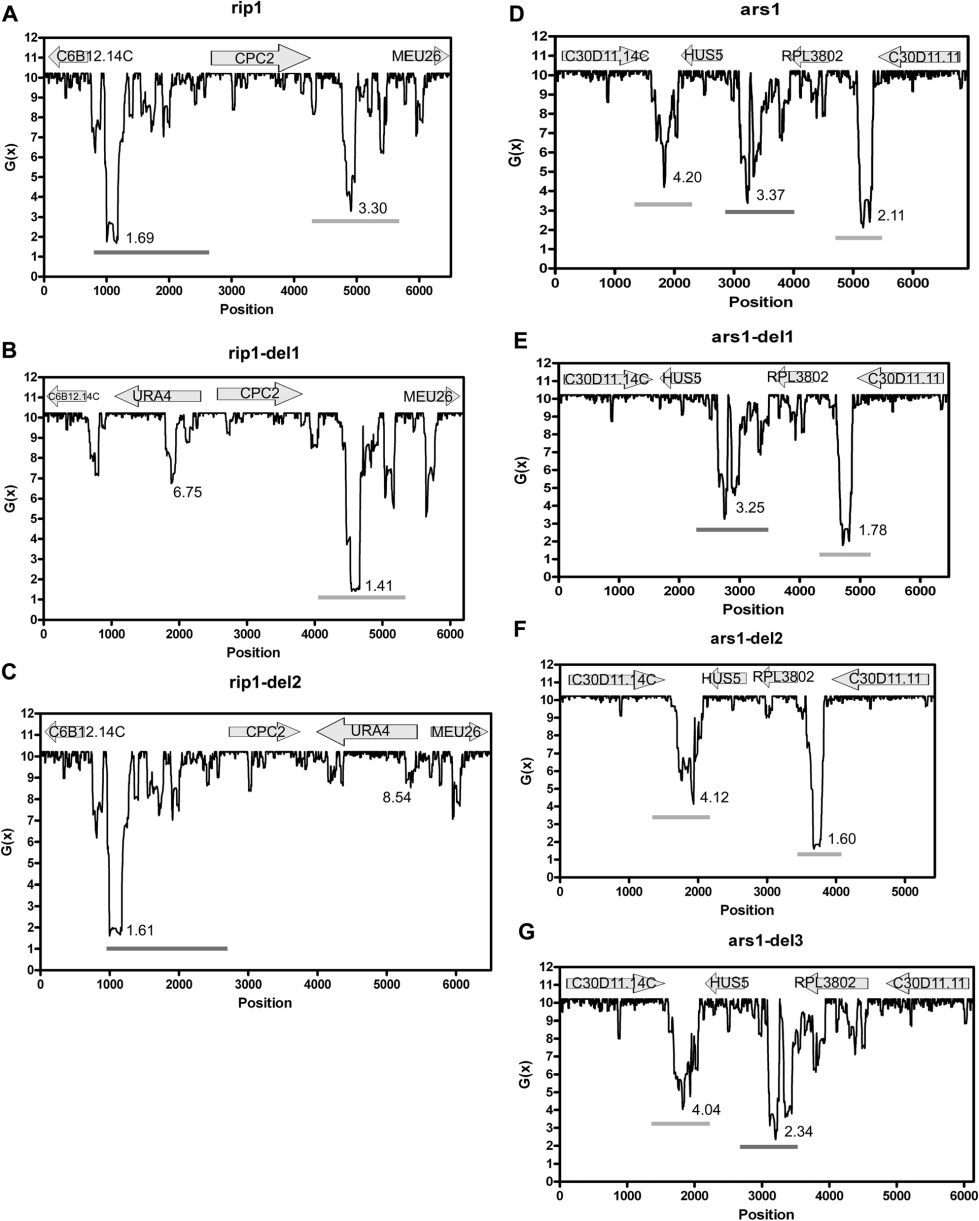
**Other closely spaced stress-destabilized intergenes show SIDD interactions akin to the *ura4 *origin region.** SIDD profiles of two ChIP-chip origins, Ori1092 (rip1) and Ori1047 (ars1), to check the interactions between the closely spaced stress destabilized intergenes following *in silico* deletions. In case of Ori1092, the intergenes were deleted *in silico* and replaced with *ura4* gene somewhat mimicking the experiments done in [18]. The locations of the origins (dark grey bar), other SIDD valleys (light grey bar) and genes (top) are shown (*S. pombe* GeneDB). The lowest *G*(x) values are mentioned below the valleys.

As mentioned above, many origin containing regions showed the presence of more than one destabilized intergenes. Are these origins similar to the *ura4* origin region in terms of interactive SIDD properties? To test this possibility, we generated *in silico* deletions of the closely spaced stress-destabilized intergenes in thirty-five randomly chosen such origins in different combinations and performed SIDD analysis of these regions. Indeed, we found a transition of SIDD valleys from one intergene to another akin to the *ura4* deletion strains in all of them. The SIDD profiles of two such origins and their deleted versions are shown in Figure 5.

## Discussion

We have analyzed SIDD profiles of different genomic regions containing three different but overlapping sets of replication origins mapped earlier by different groups, genes and non-origin intergenes of *S. pombe*. This is the first extensive SIDD profiling and comparative analysis of different genomic entities in *S. pombe.* A vast majority of origins colocalized with the IRs showing the lowest SIDD value. In fact, 99.3% of the 564 origins defined by ChIP-chip or AT content had lowest SIDD values (*G*(x) values) less than 6.07, and the four putative origins with SIDD values greater than 9 have not been verified as functional origins. It is known that the destabilization energy or the SIDD energy, *G*(x), is directly related to stability [33]. Therefore, *S. pombe* replication origins correspond to genomic regions susceptible to destabilization under stress. In fact, under the conditions of our analysis, there appears to be a gradient of stability among different genomic entities in which the origins are least stable followed by weak origins, NOIRs and the genes which are most stable.

Although the origins are more susceptible to stress-destabilization as compared to genes and non-origin intergenes in *S. pombe*, the depth of the SIDD valley alone may not be used to predict the genome-wide locations of origins because similar valleys are also present elsewhere in the genome. However, consideration of the AT-richness, a known attribute of *S. pombe* origins, makes them stand as a distinct class with the highest AT content and lowest free energy requirement for strand separation under stress. Plotting AT content versus lowest SIDD values (Figure 3B) shows a gradual increase in AT content and decrease in SIDD energy as we move from genes to NOIRs to OIRs. The lowest SIDD values and the AT contents of early- and late-firing origins [28] were found to be very similar (data not shown) suggesting no link between SIDD and the time of origin activation. Since clustered A/T stretches are essential components of fission yeast replication origins and AT-hooks of spOrc4 bind to asymmetrical As and Ts in origin regions [40-42], it appears that the binding efficiency of ORC and the extent of stress-induced destabilization both cooperatively determine origin usage and compromising with any one or both of them would adversely affect the firing frequency of an origin.

Recently, a genome-wide comparative analysis of replication origins in three species of fission yeasts, *S. pombe*, *S. octosporus* and *S. japonicus* has revealed that in contrast to AT-rich origins of *S. pombe* and *S. octosporus,* origins of *S. japonicus* are marked by the presence of GC-rich regions [43]. Preliminary results from SIDD analysis of some defined *S. japonicus* ARS elements suggest the presence of SIDD-prone DNA in them (data not shown) suggesting that even the GC-rich origins of this organism are partially stress-destabilized.

The presence of an easily unwound region at the 3^′^ end of the essential ARS consensus sequence, the binding site for the ORC proteins, is an essential feature of *S. cerevisiae* replication origins [7] and majority of them are stress-destabilized [33]. Because of a large origin size and the absence of any ACS like landmark, it would not be possible to deduce a similar relationship in case of *S. pombe* origins. However, notwithstanding the structural differences between the *S. cerevisiae* and *S. pombe* origins, SIDD appears to be a property common to both of them. This is anticipated as the presence of SIDD sites would facilitate conversion of double stranded DNA to single stranded form and ORC-assisted loading of other initiation factors on to origins. We found that the 217 ORC binding sites [28] associated with nucleosome free regions [44] are located in the same intergene, mostly within a few hundred basepairs (≤500 bp) of the SIDD sites.

It is very interesting that the earlier reported changes in the activities of the origins associated with the *ura4* origin region following their systematic deletion are paralleled by SIDD changes. We previously interpreted these results by suggesting that closely spaced origins can interfere with each other. Whichever one fires first is likely to generate replication forks that will replicate the other origins in the cluster before those other origins have a chance to fire on their own. Consequently, deleting any single origin from a cluster would be predicted to increase the firing rate of the remaining origins. Our new analyses suggest an alternative mechanism: deleting one origin in a cluster may facilitate the firing of the other origins by contributing to the SIDD of each of the other origins. Perhaps both mechanisms contribute to the observed results. Future experiments may be able to discriminate between these possibilities by comparing the effects on the functions of the origins in clusters of deletions having large SIDD effects versus deletions having small SIDD effects.

Based on the interactive behavior of the SIDD properties of the closely spaced intergenes, we predict that more than half of all the origins in *S. pombe* genome are like the *ura4* origin region having more than one closely spaced inefficient origins functioning synergistically. *In vivo* experiments would be required to find out if the observed deletion-dependent transitions of SIDD points are also followed by changes in firing frequency of the associated origins. The wide-spread alterations in SIDD properties following mutation in a DNA region [33, this study] could be utilized to study interactions between closely spaced origins (clusters of origins) in chromosomes of *S. pombe,* and probably other organisms also, to ascertain the functional organization of their replication origins.

Finally, SIDD analysis of the known origins in some other species like *Kluvyeromyces lactis*[45], *Arabidposis thaliana*[46], *Candida albicans*[47] and in humans [48-50] (see Table 3) indicates that origins are stress destabilized in these organisms also. Despite tremendous variability in their nucleotide compositions, the facilitation of duplex opening encrypted in the regulatory regions, especially origins of different genomes as shown in this and many other earlier studies [7,13,33-35], seems to be a widely conserved property. The results of this study extend similar earlier findings in *S. cerevisiae* and strongly support the idea that these properties in conjunction with other known origin attributes may be useful in predicting the locations of origins in other genomes too.

**Table 3:** The replication origins of other organisms are also stress-destabilized

**Organism (No. of proven origins)**	**Average AT%**	**Average Lowest *G*(x) value (kcal/mol)**	**Average Lowest *G*(x) value in 5 Kb context (kcal/mol)**	**Reference**
*K.lactis *(10)	65.69	1.71*	4.05	[45]
*C.albicans* (2)	67.7	1.57*	3.13	**
*A.thaliana* (4)	64.13	1.52*	5.52	[46]
Mammals (3)	53.4		2.33^#^	[48-50]

* Lowest *G*(x) value −1 kb upstream-downstream, ** Unpublished, ^#^GenBank Accession numbers of mammalian origins are M94363, Y09885, U01317.

Averages of AT% and the lowest *G*(x) values of replication origins in other organisms.

## Conclusions

SIDD analysis of replication origins and comparison regions of *S. pombe* shows that the origins are located in intergenic regions (OIRs) which are significantly more susceptible to strand separation under superhelical stress than NOIRs and genes. SIDD appears to be a widely conserved origin property that can be used to predict origin locations in conjunction with other known origin attributes, e.g., AT richness in case of *S. pombe*. The interactive nature of SIDD can also be used to predict interaction between closely-spaced origins as the deletion-induced changes in origin activity are accompanied by similar changes in degree of susceptibility to destabilization.

### Availability of supporting data

The data sets supporting the results of this article are included within the article and its additional files.

## Abbreviations

2D: Two-dimensional electrophoresis origin mapping technique; ARS: Autonomously replicating sequence; ACS: ARS core consensus sequence; NOIRs: Non-origin intergenic regions; MCM: Mini chromosome maintenance proteins; ORC: Origin recognition complex; OIRs: Origin containing intergenic regions; SIDD: Stress-induced duplex destabilization.

## Misc

Mukesh P Yadav and Sreedevi Padmanabhan equal contributors.

## Competing interests

The authors declare that they have no competing interests.

## Authors’ contributions

MPY and SP analyzed chromosome 1. MPY did the deletion analysis of the ura4 origin region and weak origins. SP did the 2D origins and the origins in other organisms, compiled the figures and tables. VPT analyzed chromosome 2 and RKM analyzed chromosome 3. DDD conceptualized the analysis, analyzed the raw data and wrote the manuscript. All authors read the final version of the manuscript and agreed upon the findings reported therein.

## Supplementary Material

Additional file 1 **Table S1.** The size, AT% and lowest *G*(x) values of all the intergenes proven as origins by 2D technique in 1-kb upstream and downstream, 5-Kb and 10-Kb windows centered on origins (OIRs). Click here for file

Additional file 2 **Table S2.** Table showing lowest *G*(x) values, size, AT% of the ChIP-chip origins and AT-islands, weak origins, genes and NOIRs analyzed in 5-kb context. Click here for file
