# Supplementary material for: Analysis of stress-induced duplex destabilization (SIDD) properties of replication origins, genes and intergenes in the fission yeast, Schizosaccharomyces pombe
Source: BMC Res Notes. 2012 Nov 19;5:643. doi: 10.1186/1756-0500-5-643 (PMC3533806; doi:10.1186/1756-0500-5-643)
Supplement: Additional file 1 — Table S1. The size, AT% and lowest G(x) values of all the intergenes proven as origins by 2D technique in 1-kb upstream and downstream, 5-Kb and 10-Kb windows centered on origins (OIRs). [file 1756-0500-5-643-S1.pdf]

Table S1 The size, AT% and lowest *G*(x) values of all the intergenes proven as origins by 2D technique in 1-kb upstream and downstream, 5-Kb and 10-Kb windows centered on origins (OIRs)

S.No.	2D / Combing proved origins	Intergene size (bp)	AT (%)	Lowest *G*(x) value (kcal/mol)			Chromosome	Reference
				**1-kb ups-downs**	**5-kb context**	**10-kb context**		
1	ori1	832	72.5	1.37	1.67	4.92	2	19
2	ori2	1052	70.4	1.63	2.48	4.68	2	
3	ori3	1919	71.1	2.56	1.43	2.39	2	
4	ori4	1279	70.5	1.33	1.56	4.22	2	
5	ori5	1926	69.1	1.68	1.74	1.74	2	
6	ars3003	2020	68.9	1.79	1.38	1.31	3	18
7	ars3002	2148	71.4	1.8	1.69	6.65	3	
8	AT1002	1387	73.2	1.47	1.81	4	2	25
9	AT1003	4860	70	1.75	1.23	1.57	2	
10	AT1015	1004	69.3	1.43	2	2.46	1	
11	AT1024	1414	72.9	1.71	1.66	1.68	1	
12	AT1041	2739	70.2	1.37	1.33	2	1	
13	AT1153	1905	69	1.3	1.41	1.75	1	
14	AT2022	2126	70.4	1.81	1.97	1.81	2	
15	AT2026	1662	72	1.31	1.53	1.94	2	
16	AT2041	1257	71.2	1.66	1.62	1.31	2	
17	AT2055	659	79	2.17	1.71	2.31	2	
18	AT2067	2135	73	1.59	1.53	2.13	2	
19	AT2072	3036	70	2.58	2.3	1.94	2	
20	AT2096	1503	71.2	1.46	2.32	1.76	2	
21	AT3043	1641	64.7	1.8	1.43	1.4	3	
22	AT3044	950	73.9	1.43	1.9	1.77	3	

23	AT3053	2170	65.5	1.21	1.95	1.71	3	
24	AT3057	2324	67.7	1.31	1.28	2.11	3	
25	ORIc7	1260	71.1	1.3	1.77	1.46	2	
26	ars2002	1017	70	1.64	1.49	2.04	2	21
27	ars3001	2171	69.6	1.34	1.56	2.42	3	23
28	3005.3	1814	66.5	1.58	1.53	5.16	3	22
29	3005.5	1056	69.3	1.66	1.67	1.85	3	
30	ORI12	1466	72.3	1.38	1.34	1.5	1	20
31	ORI76	2329	69.6	1.56	1.61	1.93	1	
32	ORI19	1620	69.3	1.32	1.59	1.65	1	
33	ORI22	1184	71.2	1.43	1.67	2.23	1	
34	ORI75	3240	69.1	1.41	1.44	2.28	2	
35	ars1	1497	68.6	1.32	1.76	5.79	1	
36	ORI18/ORIJ	1675	69.8	1.53	1.39	4	1	24
37	ORIc27	1663	70.3	1.9	1.51	1.36	1	
38	rip1	2069	68.6	1.81	1.8	6.11	1	
39	ORIc10	1263	72.4	1.5	1.35	1.24	1	
40	pcr1	1942	69.7	1.64	1.65	1.61	1	
41	tug1	1923	70.8	1.34	1.74	1.79	2	
42	rum1	2082	71.9	1.49	1.62	1.78	2	
43	ORIc11	2047	72.1	1.47	1.87	2.43	3	
44	nmt1	1571	70	1.66	1.65	3.16	3	
45	AT2014	1682	71.2	1.38	2.04	6.73	2	
46	AT2091	1913	72.5	1.58	1.59	2.31	2	
47	AT2112	1421	72.6	1.76	1.35	2.64	2	
48	AT3040	1922	72.3	1.51	1.45	0.05	3	26
	**Average**	**1787**	**70.58**	**1.58**	**1.65**	**2.56**		
